# Supplementary material for: Task-Rate-Related Neural Dynamics Using Wireless EEG to Assist Diagnosis and Intervention Planning for Preschoolers with ADHD Exhibiting Heterogeneous Cognitive Proficiency
Source: J Pers Med. 2022 Apr 30;12(5):731. doi: 10.3390/jpm12050731 (PMC9143733; doi:10.3390/jpm12050731)
Supplement: Supplementary file 1 [file jpm-12-00731-s001.zip › jpm-1613916-supplementary.pdf]

Supplementary Table 1.1 Resting relative spectral power between ADHD and TD

		Fp			Fz			Ctrl			Pz			Ocptl		
		mean	SD	p	mean	SD	p	mean	SD	p	mean	SD	p	mean	SD	p
Delta	ADHD	32.83	2.67	0.726	33.31	2.60	0.265	33.02	2.21	0.581	32.85	1.98	0.700	32.43	2.15	0.975
	TD	32.59	3.17		34.20	4.09		32.78	1.08		33.01	1.26		32.44	1.48	
Theta	ADHD	27.36	1.40	0.859	27.59	1.42	0.202	28.01	1.23	0.287	27.92	1.24	0.723	27.53	1.29	0.951
	TD	27.30	1.61		28.07	1.79		27.74	0.70		28.01	0.79		27.51	0.80	
Alpha	ADHD	23.15	0.75	0.776	23.06	0.89	0.112	23.04	0.68	0.636	23.11	0.97	0.135	23.32	0.80	0.193
	TD	23.22	1.37		22.63	1.39		22.97	0.49		22.82	0.49		23.09	0.62	
Beta	ADHD	16.67	3.41	0.265	16.03	3.28	0.308	15.93	2.71	0.278	16.11	2.23	0.932	16.72	2.70	0.681
	TD	16.90	3.46		15.10	4.49		16.51	1.24		16.15	1.49		16.95	1.70	

ADHD=ADHD-H+ADHD-L(n=42), TD (n=31)

Supplementary Table 1.2 Resting relative spectral power among three groups

		Fp			Fz			Ctrl			Pz			Ocptl		
		mean	SD	p	mean	SD	p	mean	SD	p	mean	SD	p	mean	SD	p
Delta	ADHD-H	32.97	2.97	0.879	32.98	2.34	0.406	33.09	2.58	0.831	32.50	1.94	0.283	32.49	2.52	0.969
	ADHD-L	32.64	2.27		33.76	2.91		32.94	1.66		33.32	1.99		32.34	1.61	
	TD	32.59	3.17		34.20	4.09		32.78	1.08		33.01	1.26		32.44	1.48	
Theta	ADHD-H	27.28	1.57	0.908	27.42	1.49	0.325	27.99	1.36	0.564	27.79	1.19	0.622	27.57	1.46	0.959
	ADHD-L	27.47	1.16		27.82	1.32		28.03	1.07		28.10	1.33		27.47	1.05	
	TD	27.30	1.61		28.07	1.79		27.74	0.70		28.01	0.79		27.51	0.80	
Alpha	ADHD-H	23.15	0.86	0.961	23.23	0.80	0.157	23.09	0.73	0.737	23.25	0.95	0.141	23.36	0.90	0.388
	ADHD-L	23.15	0.59		22.84	0.98		22.97	0.63		22.92	0.99		23.26	0.65	
	TD	23.22	1.37		22.63	1.39		22.97	0.49		22.82	0.49		23.09	0.62	
Beta	ADHD-H	16.60	3.79	0.951	16.37	3.25	0.481	15.84	3.16	0.528	16.46	2.18	0.419	16.57	3.13	0.819
	ADHD-L	16.75	2.93		15.58	3.34		16.06	2.02		15.66	2.27		16.92	2.08	
	TD	16.90	3.46		15.10	4.49		16.51	1.24		16.15	1.49		16.95	1.70	

ADHD-H (n=24), ADHD-L (n=18), TD(n=31)

Supplementary Table 2.1 Slow-rate task relative spectral power between ADHD and TD

		Fp			Fz			Ctrl			Pz			Ocptl		
		mean	SD	p	mean	SD	p	mean	SD	p	mean	SD	p	mean	SD	p
Delta	ADHD	33.56	2.66	0.180	33.98	2.02	0.666	33.56	1.29	**0.012**	33.60	1.09	**0.030**	32.94	1.75	0.350
	TD	32.60	3.40		34.42	6.09		32.77	1.28		32.97	1.35		32.36	1.54	
Theta	ADHD	27.61	1.62	0.476	27.80	1.25	0.381	27.96	0.99	0.235	28.18	1.01	0.407	27.94	1.25	0.972
	TD	27.32	1.80		28.19	2.45		27.71	0.75		27.99	0.86		27.70	0.76	
Alpha	ADHD	22.78	0.91	0.190	22.62	0.70	0.604	22.62	0.46	**0.045**	22.50	0.51	0.289	22.86	0.72	0.084
	TD	23.15	1.46		22.44	2.15		22.87	0.57		22.63	0.55		22.86	0.57	
Beta	ADHD	16.05	3.33	0.296	15.59	2.65	0.559	15.86	1.69	**0.039**	15.72	1.42	0.067	16.26	2.18	0.532
	TD	16.93	3.80		14.96	6.36		16.66	1.48		16.41	1.73		17.08	1.72	

ADHD=ADHD-H+ADHD-L(n=42), TD (n=31), bold indicated significance p<0.05

Supplementary Table 2.2 Slow-rate task relative spectral power among three groups

		Fp			Fz			Ctrl			Pz			Ocptl		
		mean	SD	p	mean	SD	p	mean	SD	p	mean	SD	p	mean	SD	p
Delta	ADHD-H	33.52	3.08	0.409	33.75	1.79	0.843	33.73	1.38	**0.026**	33.32	0.81	**0.022**	33.16	1.97	0.211
	ADHD-L	33.60	2.07		34.28	2.32		33.33	1.16		33.97	1.31		32.65	1.41	
	TD	32.60	3.40		34.42	6.09		32.77	1.28		32.97	1.35		32.36	1.54	
Theta	ADHD-H	27.57	1.73	0.764	27.73	1.41	0.658	28.06	0.94	0.352	28.07	0.97	0.512	27.98	1.30	0.625
	ADHD-L	27.67	1.52		27.89	1.04		27.83	1.07		28.32	1.08		27.89	1.23	
	TD	27.32	1.80		28.19	2.45		27.71	0.75		27.99	0.86		27.70	0.76	
Alpha	ADHD-H	22.84	1.11	0.397	22.78	0.61	0.646	22.61	0.50	0.134	22.65	0.51	**0.054**	22.82	0.76	0.899
	ADHD-L	22.70	0.57		22.42	0.77		22.64	0.42		22.30	0.45		22.92	0.69	
	TD	23.15	1.46		22.44	2.15		22.87	0.57		22.63	0.55		22.86	0.57	
Beta	ADHD-H	16.07	3.68	0.582	15.73	2.68	0.824	15.60	1.72	**0.059**	15.96	1.28	**0.101**	16.04	2.49	0.164
	ADHD-L	16.03	2.90		15.41	2.68		16.20	1.64		15.41	1.57		16.55	1.72	
	TD	16.93	3.80		14.96	6.36		16.66	1.48		16.41	1.73		17.08	1.72	

ADHD-H (n=24), ADHD-L (n=18), TD(n=31), bold indicated significance (p<0.05) in the post-hoc analysis

Supplementary Table 3.1 Fast-rate task relative spectral power between ADHD and TD

		Fp			Fz			Ctrl			Pz			Ocptl		
		mean	SD	p	mean	SD	p	mean	SD	p	mean	SD	p	mean	SD	p
Delta	ADHD	33.37	3.27	0.532	34.07	2.80	0.564	33.66	1.76	0.153	33.50	1.34	0.599	32.73	1.59	0.563
	TD	32.86	3.64		34.67	5.86		33.13	1.22		33.34	1.25		32.52	1.47	
Theta	ADHD	27.62	1.82	0.820	27.84	1.45	0.227	28.11	1.04	0.399	28.22	1.02	0.686	27.96	1.09	0.797
	TD	27.52	1.85		28.38	2.31		27.93	0.70		28.30	0.79		27.90	0.79	
Alpha	ADHD	22.96	1.21	0.536	22.72	1.06	0.439	22.57	0.58	0.131	22.54	0.69	0.978	22.94	0.77	0.615
	TD	23.16	1.52		22.43	2.04		22.79	0.65		22.55	0.50		22.86	0.60	
Beta	ADHD	16.05	3.88	0.659	15.37	3.28	0.446	15.66	2.26	0.276	15.74	1.60	0.851	16.37	1.91	0.413
	TD	16.46	3.98		14.52	6.12		16.15	1.27		15.81	1.48		16.72	1.69	

ADHD=ADHD-H+ADHD-L(n=42), TD (n=31)

Supplementary Table 3.2 Fast-rate task relative spectral power among three groups

		Fp			Fz			Ctrl			Pz			Ocptl		
		mean	SD	p	mean	SD	p	mean	SD	p	mean	SD	p	mean	SD	p
Delta	ADHD-H	33.06	3.87	0.653	33.45	2.79	0.489	33.51	2.19	0.281	32.92	1.30	**0.002**	32.58	1.75	0.657
	ADHD-L	33.79	2.26		34.89	2.67		33.86	0.95		34.27	0.96		32.93	1.37	
	TD	32.86	3.64		34.67	5.86		33.13	1.22		33.34	1.25		32.52	1.47	
Theta	ADHD-H	27.47	2.02	0.804	27.63	1.62	0.339	28.06	1.21	0.645	27.95	1.09	0.090	27.80	1.16	0.484
	ADHD-L	27.83	1.56		28.13	1.18		28.18	0.78		28.57	0.80		28.16	0.99	
	TD	27.52	1.85		28.38	2.31		27.93	0.70		28.30	0.79		27.90	0.79	
Alpha	ADHD-H	23.05	1.50	0.728	22.93	1.16	0.436	22.62	0.70	0.269	22.76	0.79	**0.030**	23.02	0.84	0.656
	ADHD-L	22.84	0.68		22.43	0.86		22.51	0.37		22.26	0.39		22.85	0.68	
	TD	23.16	1.52		22.43	2.04		22.79	0.65		22.55	0.50		22.86	0.60	
Beta	ADHD-H	16.42	4.43	0.704	15.98	3.43	0.468	15.81	2.87	0.467	16.37	1.81	**0.007**	16.60	2.19	0.463
	ADHD-L	15.55	3.03		14.55	2.96		15.46	1.04		14.90	0.66		16.06	1.45	
	TD	16.46	3.98		14.52	6.12		16.15	1.27		15.81	1.48		16.72	1.69	

ADHD-H (n=24), ADHD-L (n=18), TD(n=31), bold indicated significance (p<0.05) in the post-hoc analysis
